# Inhibitor of DNA binding 2 (Id2) mediates microtubule polymerization in the brain by regulating αK40 acetylation of α-tubulin

**DOI:** 10.1038/s41420-021-00652-4

**Published:** 2021-09-21

**Authors:** Taegwan Yun, Hyo Rim Ko, Dong-Gyu Jo, Kye Won Park, Sung-Woo Cho, Jihoe Kim, Jee-Yin Ahn

**Affiliations:** 1 Department of Molecular Cell Biology, University School of Medicine, 16419 Suwon, Korea; 2 Single Cell Network Research Center Sungkyunkwan, University School of Medicine, 16419 Suwon, Korea; 3 Department of Health Science and Technology, Samsung Advanced Institute for Health Science and Technology, Sungkyunkwan University, 06351 Seoul, Korea; 4 School of Pharmacy, Sungkyunkwan University, 16419 Suwon, Korea; 5 Department of Food Science and Biotechnology, Sungkyunkwan University, 16419 Suwon, Korea; 6 Department of Biochemistry and Molecular Biology, University of Ulsan, College of Medicine, 05505 Seoul, Korea; 7 Department of Medical Biotechnology, Yeungnam University, 38541 Gyeongsan, Republic of Korea; 8 Samsung Biomedical Research Institute, Samsung Medical Center, 06351 Seoul, Korea

**Keywords:** Microtubules, Phosphorylation

## Abstract

Acetylation of α-tubulin lysine 40 (αK40) contributes to microtubule (MT) stability and is essential for neuronal development and function, whereas excessive αK40 deacetylation is observed in neurodegenerative disorders including Alzheimer’s disease (AD). Here we identified inhibitor of DNA binding 2 (Id2) as a novel MT-binding partner that interacts with α-tubulin and enhances αK40 acetylation, leading to MT polymerization in the neurons. Commensurate with our finding that the low levels of Id2 expression along with a reduced αK40 acetylation in the postmortem human AD patient and 5X-FAD, AD model mice brain, Id2 upregulation in the hippocampus of 5X-FAD, which exhibit high levels of Sirt2 expression, increased αK40 acetylation and reconstitutes axon growth. Hence our study suggests that Id2 is critical for maintaining MT stability during neural development and the potential of Id2 to counteract pathogenic Sirt2 activity in AD.

## Introduction

Inhibitor of DNA binding 2 (Id2) is a negative regulator of basic helix–loop–helix (bHLH) transcription factors. During development, Id2 binding to bHLH transcription factors suppresses the expression of several growth inhibitory molecules, promoting axon growth [1]. Id2 degradation in the brain by the anaphase-promoting complex/cyclosome and its activator Cdh1 (APC/C^Cdh1^) reduces E protein-dependent axonal growth, thus maintaining axon morphology [2, 3], while Id2 protection results in erratic growth and abnormal distribution of parallel fibers in the cerebral cortex [4]. We have recently demonstrated that Id2 phosphorylation at serine 14 (S14) by Akt enhances axonal growth and growth cone formation in developing neurons by controlling actin-cytoskeleton dynamics [5, 6].

Neuronal morphology and migration are controlled by the MT dynamics [7]. Acetylation of α-tubulin lysine 40 (αK40) has received particular interest because it is the only tubulin post-translational modification (PTM) found in the MT lumen and is known to confer stability [8, 9]. Loss of MT stability may contribute to pathology as αK40 acetylation is reduced in neurodegenerative disorders, including Alzheimer’s disease (AD) [10, 11], Huntington’s disease (HD) [12], and Parkinson’s disease (PD) [13]. The role of αK40 acetylation has been obtained mainly by manipulating the αK40 acetyltransferase αTAT1/MEC-17 or the deacetylases Sirt2 and HDAC6. For instance, a nematode αTAT1 mutant demonstrated profound MT defects [14]. Similarly, modulation of Sirt2, the predominant isoform in the brain, profoundly affects neurite outgrowth and synapse remodeling [15–17]. Mice without Sirt2 exhibited axonal degeneration and locomotor disability [18]. In AD, Sirt2 is abnormally overexpressed and thus excessively deacetylates tubulin, which results in MT destabilization and tau dissociation [19]. HDAC6 is another major α-tubulin deacetylase in some cells [20, 21], but tubulin is not hyperacetylated in the brains of HDAC6 deficient mice [22].

Here, we demonstrate that Id2 is a novel tubulin-binding partner and potent regulator of MT dynamics in neurons. Id2 promotes α-tubulin polymerization and enhanced αK40 acetylation by competing for tubulin access with Sirt2. In postmortem brain of AD patients and hippocampus of AD model (5X-FAD) mice, we found that notably diminished levels of Id2 compared to age-matched control, implying the reduction of Id2 is correlated with impaired MT stability. Reconstitution of Id2-WT but not Id2-S14A, a phosphor-ablated mutant, into the hippocampus of 5X-FAD mice efficiently augmented αK40 acetylation and improves axon growth, suggesting possible beneficial functions of Id2 in neurodegenerative disorders characterized by MT instability such as AD.

## Results

### Id2 interacts with α-tubulin in the developing brain

**Fig. 1:**
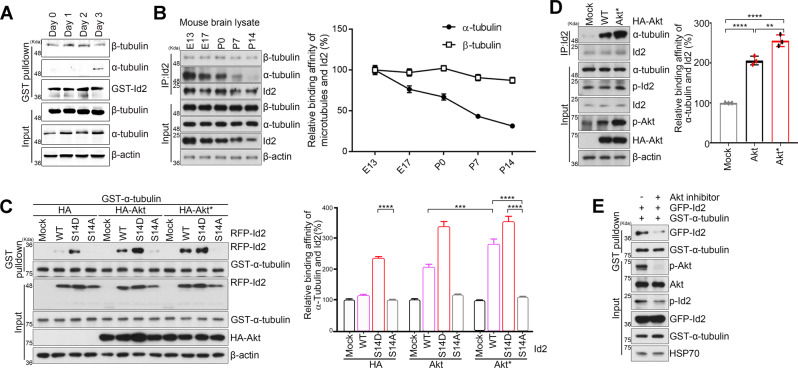
Id2 interacts with α-tubulin in the developing brain. **A** Purified GST–Id2 was reacted with PC12 cell lysate and immunoblotting was performed with antibodies as indicated. **B** Mouse brain lysates were subjected to immunoprecipitation with anti-Id2 antibody and analyzed by immunoblotting. **C** GST–α-tubulin was transfected into HEK293T cells with RFP–Id2 WT, S14D, or S14A, and HA–Akt WT or constitutively active (CA) Akt. Cell lysates were subjected to GST pull-down and analyzed by immunoblotting. **D** HA–Akt-WT or CA-Akt transfected HEK293T cells were applied to immunoprecipitation and immunoblotting as indicated. Bar graph shows α-tubulin-binding quantification. **E** GFP–Id2 and GST–α-tubulin transfected cells were treated with Akt inhibitor VIII (1 μM) for 6 h and conducted GST pull-down analysis. **p < 0.005, ***p < 0.0005, ****p < 0.0001. All values are mean ± SEM and the representative images shown are from at least three independent experiments.

Because Id2 localized both at the peripheral domain of growth cone, binding to F-actin when phosphorylated by Akt and at the central domain of axon shaft [5, 6], we speculated that Id2 may contribute to the regulation of MT dynamics by cross-linking the actin-cytoskeleton. Although Id2 interacts with both α- and β-tubulin, the interaction between Id2 and β-tubulin was constant, but the interaction between Id2 and α-tubulin was augmented during PC12 cell differentiation (Fig. 1A). Accordingly, Id2 preferentially interacts with α-tubulin during embryonic development and that this interaction gradually decreases after birth. The association of Id2 and β-tubulin was constant throughout the observation period (Fig. 1B). Thus, Id2 interacts with α-tubulin in a regulated manner and that this interaction may contribute to neural development. Therefore, we focused on the association of Id2 and α-tubulin.

In developing neurons, Id2-S14 phosphorylation by Akt is essential for the augmentation of axon growth [5]. Intriguingly, the phospho-mimetic mutant form of Id2 (S14D) showed enhanced association with α-tubulin, whereas the S14A phospho-ablated mutant barely interacted with α-tubulin compared to WT Id2. The interaction between WT Id2 and α-tubulin was slightly increased by Akt overexpression, and the strongest interaction occurred in the presence of CA Akt (Fig. 1C). Moreover, the endogenous interaction between Id2 and α-tubulin was stronger in the presence of CA Akt compared to WT Akt (Fig. 1D). Conversely, Akt inhibitor VIII treatment markedly reduced the interaction of Id2 and α-tubulin (Fig. 1E). These data suggest that S14 phosphorylation on Id2 enhances the interaction with α-tubulin.

### Id2 promotes α-tubulin incorporation into microtubules and microtubule stabilization

**Fig. 2:**
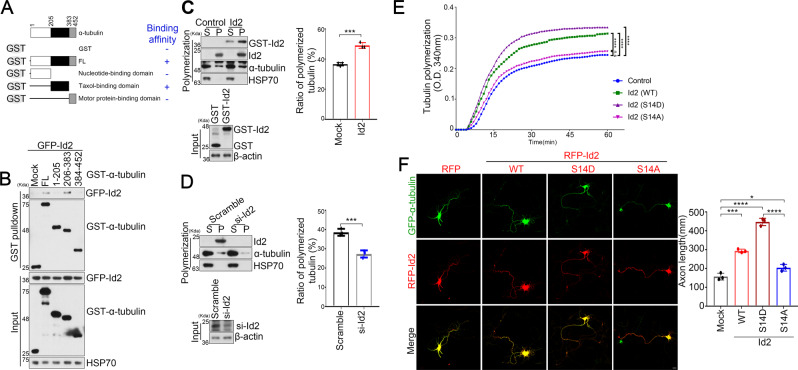
Id2 promoted α-tubulin incorporation into polymers and microtubule stabilization. **A**, **B** HEK293T cells were co-transfected with GFP–Id2 and mammalian GST–α-tubulin fragments (**A**). Cell lysates were subjected to GST pull-down analysis. **C**, **D** Cells were transfected with GST–Id2 (**C**) or si-Id2 (**D**), respectively, and lysates (pellets containing insoluble polymerized tubulin and supernatants containing soluble tubulin dimer) were subjected to in vitro tubulin polymerization assays. Densitometry analysis shows the percent ratio of polymerized tubulin (right). **E** Tubulin proteins (>99% purity) were suspended in reaction buffer in the presence of purified GST–Id2 plasmids. The MT polymerization was measured absorbance at 340 nm. **F** Cultured neurons were co-transfected with GFP–α-tubulin and RFP–Id2 WT, S14A, S14D, or vector control on DIV 3 and fixed on DIV 5. Quantification of axon length measurements from three independent experiments is shown on the right panel. Scale bar, 20 μm. *p* > 0.05, *p < 0.05, ***p < 0.0005, ****p < 0.0001. All values are mean ± SEM and the representative images shown are from at least three independent experiments.

As our domain mapping analysis showed that specific interaction of I2 and the intermediate domain of α -tubulin, a segment also essential for the binding of the MT-stabilizing agent and clinical anticancer drug taxol [23] (Fig. 2A, B), we hypothesized that Id2 binding to α-tubulin may regulate microtubule polymerization and/or stabilization. To test this hypothesis, we conducted in vitro cell-based tubulin polymerization assays. Microtubule polymers (P) accounted for 35% of total tubulin mass in control cells but 50% in cells overexpressing Id2 (Fig. 2C). In contrast, depletion of Id2 reduced MT polymer mass from 40 to 25% of the total, and almost all α-tubulin partitioned in the MT-unbound supernatant fraction (Fig. 2D). Moreover, Id2-WT or S14D enhanced tubulin polymerization whereas S14A mutant, which is not able to bind to α-tubulin, showed a similar blotting pattern as the control (Fig. 2E and Supplementary Fig. 1), implying that the binding of Id2 to α-tubulin is essential for MT polymerization and stability. Consistent with previous reports those Id2 is an unstable protein but Akt mediated S14 phosphorylation protects Id2 from degradation [3, 5], the purified GST-S14A mutant is relatively unstable compared with WT or S14D, displaying more evident degradation during GST-fusion protein purification (Supplementary Fig. 1).

To explore the functional relevance of the association of Id2 and α-tubulin in developing neurons, we co-transfected GFP–α-tubulin and RFP–Id2 (WT, S14D, and S14A) into hippocampal neurons. Overexpression of WT Id2 increased axon growth by around twofold, and Id2 S14D expression further enhanced axon growth. However, neurons transfected with the S14A mutant exhibited a substantially shorter axon compared to neurons expressing Id2 WT or Id2 S14D but similar to that of control (Fig. 2F). Taken together, these data imply that Id2 contributes to microtubule polymerization, thereby enhancing axon growth.

### Id2 augments acetylation of α-tubulin and enhances MT stabilization

**Fig. 3:**
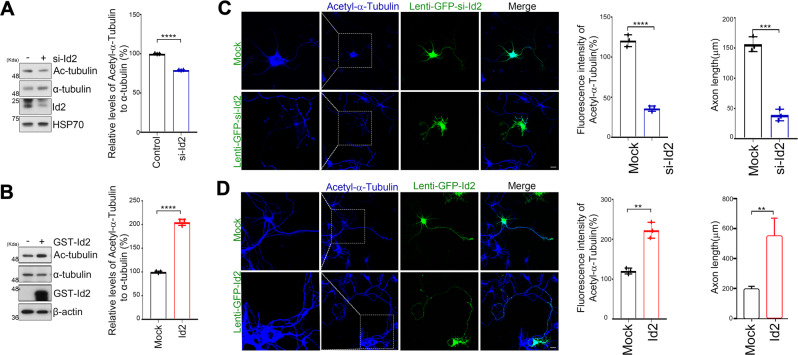
Id2 augments acetylation of α-tubulin and enhances MT stabilization. **A**, **B** Si-Id2 (**A**) or GST–Id2 (**B**) transfected, hippocampal neurons were subjected to immunoblotting with the indicated antibodies. Quantification of acetyl-α-tubulin is shown as a bar graph (right). **C**, **D** Hippocampal neurons were infected with pLenti-GFP–si-Id2 and control (**C**), or pLenti-GFP–Id2 and control (**D**) on DIV 3 and fixed on DIV 5. Neurons were stained with anti-acetyl-α-tubulin (blue). Representative images with higher magnification of the region indicated by a box show acetylated tubulin immunofluorescence. Quantification of fluorescence intensity and axon length is shown on the right. Scale bar, 20 μm. **p < 0.005, ***p < 0.0005, ****p < 0.0001. All values are mean ± SEM and the representative images shown are from at least three independent experiments.

Acetylation of α-tubulin lysine 40 (αK40) is a critical PTM for MT mechanical stability; therefore, disruption of this process leads to MT instability and axon degeneration [14, 24]. Given that Id2 binding to α-tubulin is required for MT stability and polymerization, we determined whether Id2 is involved in the regulation of αK40 acetylation during neural development. Indeed, Id2 knockdown elicited a ~20% reduction in αK40 acetylation while Id2 overexpression resulted in a robust increase in αK40 acetylation compared with control hippocampal neurons (Fig. 3A, B).

To further confirm the effect of Id2 on αK40 acetylation, we conducted αK40 immunofluorescence staining in developing hippocampal neurons. Consistently, immunoreactivity of acetyl-α-tubulin was attenuated in GFP-lentiviral-Id2 siRNA expressing neurons and these neurons exhibited many short axons, whereas GFP-lentiviral-Id2 expressing neurons visualized markedly intense acetyl-α-tubulin immunostaining as well as much longer axons compared to control GFP-lentivirus-expressing neurons (Fig. 3C, D). Thus, Id2 promotes αK40 acetylation, which in turn contributes to the stabilization of MTs for proper axonal growth.

### Id2 protects αK40 acetylation by blocking Sirt2-mediated deacetylation

**Fig. 4:**
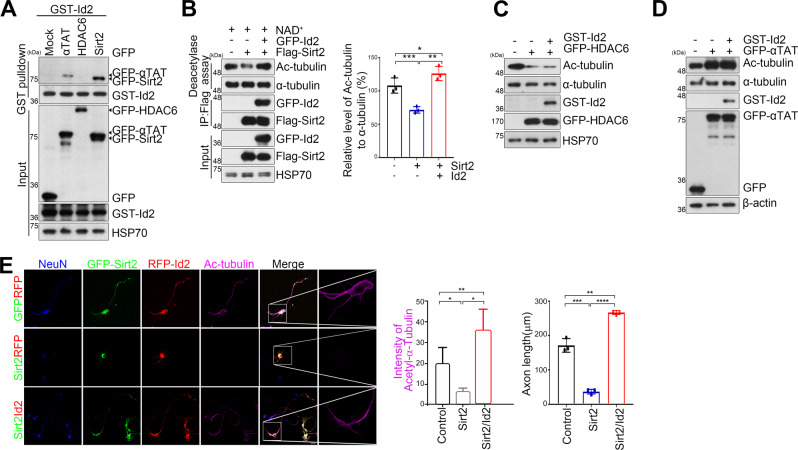
Id2 protects αK40 acetylation by blocking Sirt2-mediated deacetylation. **A** HEK293T cells were transfected as indicated and the cell lysates were subjected to GST pull-down and immunoblotting as indicated. **B** In vitro deacetylase assay was performed with GFP–Id2 and Flag–Sirt2 proteins. The bar graphs represent the quantification of the acetyl-α-tubulin to α-tubulin ratio. **C**, **D** GST–Id2 and GFP–HDAC6 (**C**) or GFP–αTAT (**D**) transfected cell lysates were subjected to immunoblotting as indicated. **E** Primary cultured neurons were co-transfected with GFP–Sirt2 or GFP vector control and RFP–Id2 or RFP vector on DIV 3 and fixed on DIV 5. Neurons were stained with anti-NeuN (blue) and anti-acetylated tubulin (purple). Quantification of fluorescence intensity and axon length is shown at the right. Scale bar, 20 μm. *p < 0.05, **p < 0.005, ***p < 0.0005, ****p < 0.0001. All values are mean ± SEM and the representative images shown are from at least three independent experiments.

The level of α-tubulin acetylation is governed by the opposing actions of αTAT1 acetyltransferase and Sirt2 and/or HDAC6 deacetylases. Based on our finding that Id2 augmented acetylation of α-tubulin, we examined if Id2 promotes acetylation per se or suppresses the deacetylation of α-tubulin by physically interacting with αTAT1 or Sirt2/HDAC6. Interestingly, Id2 strongly interacts with Sirt2 but not HDAC6, and Id2 also slightly interacted with αTAT (Fig. 4A). To determine whether this Id2 interaction to deacetylase or acetyltransferase plays and roles in α-tubulin acetylation, we first compared the degree of α-tubulin deacetylation by Sirt2 in the presence or absence of Id2. Intriguingly, this deacetylation of α-tubulin was dramatically recovered by Id2 overexpression (Fig. 4B), whereas HDAC6-dependent deacetylation of α-tubulin was not altered by Id2 overexpression (Fig. 4C). However, we did not detect a significant increase of αTAT-mediated α-tubulin acetylation in the presence of Id2 (Fig. 4D), reflecting the strong binding of Id2 to Sirt2 but not to HDAC6 or αTAT. These results suggest that Id2 could promote αK40 acetylation by preventing Sirt2-mediated deacetylation.

We next accessed the physiological influence of Id2-mediated repression of Sirt2 on αK40 in developing neurons. Sirt2 overexpression dramatically abrogated αK40 acetylation and reduced axon growth. Notably, neurons expressing Sirt2 with Id2 displayed higher levels of αK40 acetylation and longer axons than controls (DIV 5) (Fig. 4E), suggesting that Id2 prevents Sirt2-mediated αK40 deacetylation and that maintained αK40 acetylation is required for normal axonal growth.

### Id2 competitively interrupts the association of Sirt2 with α-tubulin

**Fig. 5:**
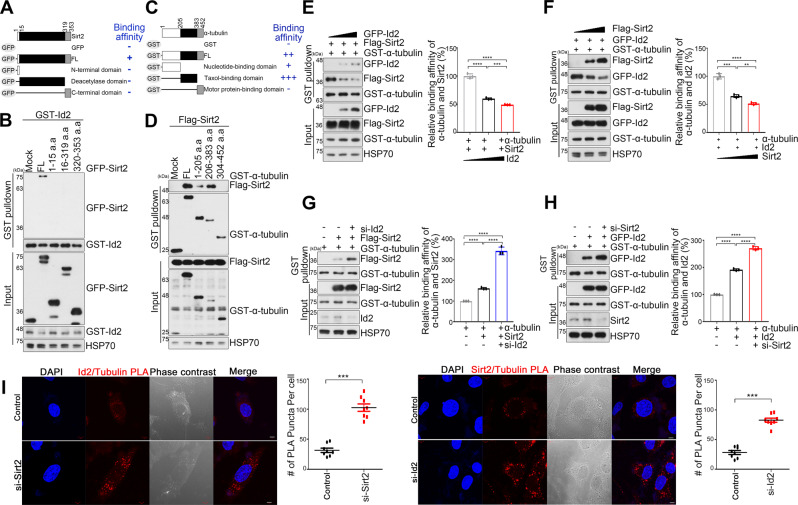
Id2 competitively interrupts the association of Sirt2 with α-tubulin. **A**, **B** GST–Id2 and mammalian GFP–Sirt2 fragments (**A**) transfected cells were subjected to GST pull-down assay and immunoblotting as indicated. **C**, **D** Flag–Sirt2 and mammalian GST–α-tubulin fragments (**C**) transfected cells were subjected to GST pull-down and immunoblotting as indicated. **E**, **F** HEK293T cells were transfected with 0, 1, 3 μg of GFP–Id2 (**E**) or Flag–Sirt2 (**F**) with GST–α-tubulin, and lysates were subjected to GST pull-down and immunoblotting. The bar graphs represent the quantification of Sirt2 or Id2–α-tubulin binding. **G**, **H** Primary hippocampal neurons were transfected as indicated and the cell lysates were subjected to GST pull-down assay. **I** Proximity ligation assay (PLA) was conducted with scr, si-Id2, or si-Sirt2 expressing cells. Confocal images were shown tubulin-Id2 PLA staining (left) and tubulin-Sirt2 PLA staining (right). Nuclei were stained by DAPI (blue). Scale bar 5 μm. Quantification of tubulin-Id2 or tubulin-Sirt2 PLA puncta is shown as a bar graph. **p < 0.005, ***p < 0.0005, ****p < 0.0001. All values are mean ± SEM and the representative images shown are from at least three independent experiments.

To investigate the molecular basis for this Id2-mediated reduction in Sirt2-dependent αK40 deacetylation, we investigated the specificity of the physical interaction between Id2 and Sirt2. Only full-length Sirt2 but not truncated forms associated with Id2, indicating that the intact three-dimensional conformation is critical for Sirt2–Id2 interaction (Fig. 5A and B). Noticeably, Sirt2 strongly bound to the intermediate domain (taxol-binding domain) within α-tubulin (Fig. 5C, D), where it interacts with Id2 (Fig. 2B), implicating that Id2 probably competes with Sirt2 for binding to the α-tubulin intermediate domain.

To assess the impact of Id2 on the interaction of Sirt2 with α-tubulin, we monitored whether Id2 and Sirt2 compete for the binding to α-tubulin. Indeed, the binding of Sirt2 to α-tubulin was progressively reduced as Id2 expression increased while the interaction between Id2 and α-tubulin was notably reduced by increased Sirt2 expression (Fig. 5E, F). Under Id2 knockdown, the binding of Sirt2 to α-tubulin increased whereas depletion of Sirt2 knockdown increased the binding of Id2 to α-tubulin in the hippocampal neurons (Fig. 5G, H). Utilizing the proximity ligation assay (PLA) to detect protein complexes, we quantified the interaction of Id2-α-tubulin or Sirt2-α-tubulin. Id2-α-tubulin PLA puncta are increased in the absence of Sirt2 compared with scramble transfected cells whereas Si-Id2 transfected cells showed a surprising increase in Sirt2-α-tubulin PLA puncta (Fig. 5I; Supplementary Fig. 2A and B). Collectively, these data strongly suggest that Id2 reduces Sirt2-mediate deacetylation of α-tubulin by competing for the same interaction site, consequently shifting the balance toward α-tubulin acetylation.

### Id2 enhances α-tubulin acetylation in the brain of Alzheimer’s disease (AD) model

**Fig. 6:**
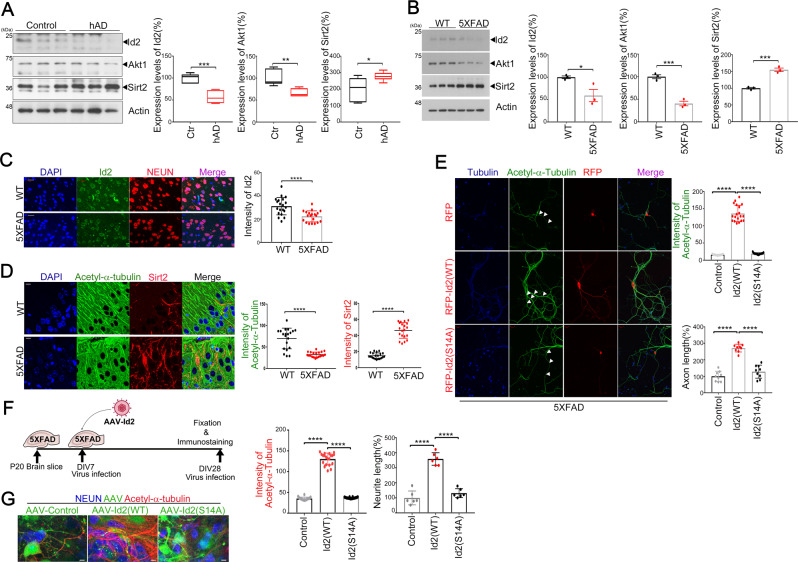
Id2 enhances α-tubulin acetylation in the brain of Alzheimer’s disease (AD) model. **A**, **B** Protein levels in postmortem brain samples from AD patients and age-matched control (each *n* = 3) (**A**) and 5X-FAD and age-matched WT mice (each *n* = 3) (**B**) were determined by immunoblotting against anti-Id2, Akt1, Sirt2, and Tubulin antibodies. **C**, **D** The paraffin-embedded sections from the hippocampus of WT or 5X-FAD mice were immunostained with anti-NEUN (red) or anti-Id2 (green) (**C**) and with anti-Sirt2 (red) or anti-acetyl-α-tubulin (green) (**D**) antibodies, and DAPI (blue), respectively. Quantification of immunoreactivity for Id2 (**C**) or acetyl-α-tubulin and Sirt2 (**D**) is shown right (mean ± SEM of 12–18 sections from *n* = 4 mice per group). Scale bar 20 μm (**C**) and 10 μm (**D**). **E** Hippocampal neurons from 5X-FAD or WT mice were transfected with RFP-Id2-WT or S14A at DIV 14. Immunostaining was performed with anti-acetyl-α-tubulin (green) and Tubulin (blue). Scale bar 20 μm. Quantification of fluorescence intensity and axon length from more than three independent experiments is shown at the right. **F**, **G** 5X-FAD brain (P20) slice cultures were performed and maintained for 28 days. The slices were infected with AAV2-Id2 WT, S14A, or control at DIV 7 and maintained for an additional 20 days. Immunostaining was conducted with anti-acetyl-α-tubulin (red) and anti-NEUN (blue). Scale bar 5 μm. *p < 0.05, **p < 0.005, ***p < 0.0005, ****p < 0.0001 versus indicated. All values are mean ± SEM and the representative images shown are from at least three independent experiments.

Microtubule disassembly driven by a reduction in tubulin acetylation has long been recognized in the AD brain [10, 25] and AD is associated with both increased Sirt2 levels and reduced decreased αK40 acetylation [19]. To define the pathological relevance of inverse correlation of Id2 levels and Sirt2 function, we employed the postmortem brain of human AD patients. Intriguingly, Id2 levels, as well as Akt levels, were notably alleviated whereas Sirt2 expression was increased in the brain of AD patients compared to age-matched control brains (Fig. 6A). We also found that Sirt2 expression is inversely correlated with Id2 and Akt levels in 5X-FAD brains (Fig. 6B). Both 5X-FAD and WT mouse brain tissues were stained against anti-Aβantibody to confirm AD pathology (Supplementary Fig. 3A). Correspondingly. Id2 levels were lower in the hippocampus of 5X-FAD compared to age-matched WT mice (4 months) (Fig. 6C). Moreover, we also found that a high level of Sirt2 expression and a low level of αK40 acetylation with distorted MT arrangement in 5X-FAD hippocampus while WT mice showed relatively well-organized MT with high αK40 acetylation and low level of Sirt2 (Fig. 6D; Supplementary Fig. 3B).

To explore the pathophysiological consequences of high Sirt2 expression and low Id2 expression, we examined whether Id2 overexpression in the hippocampus of 5X-FAD brain could attenuate Sirt2 levels, enhance αK40 acetylation. Forced expression of RFP-Id2 into hippocampal neurons of 5X-FAD prominently enhanced αK40 acetylation, promoting axon growth compared to RFP vector-expressing neurons. In contrast, RFP-Id2-S14A expressing neurons did not redeem axon growth and αK40 acetylation (Fig. 6E; Supplementary Fig. 3C). Although the effect was not dramatic that is shown in 5X-FAD neurons, overexpression of Id2 WT but not Id2-S14A slightly increased αK40 acetylation and enhanced axon growth in the hippocampal neurons of WT mice (Supplementary Fig. 3D). We identified axon as the longest neurite that is stain with anti-tau antibody or anti-Id2 antibody as Id2 is co-localized with tau in the growing axon (Supplementary Fig. 3E). In addition, we assessed ex vivo organotypic hippocampus slice cultures from WT and 5X-FAD mice (Fig. 6F). To our surprise, Id2 WT but not Id2 S14A dramatically augmented αK40 acetylation in the hippocampal CA3 region of 5X-FAD mice and promoted neurite growth reflecting MT dynamics compared to controls (Fig. 6G; Supplementary Fig. 4A and B). Taken together, our data indicate that Id2 protects α-tubulin from Sirt2-mediated deacetylation, and maintaining αK40 acetylation by reconstitution of Id2 signaling improves MT dynamics in AD brain.

## Discussion

Here, we demonstrate that Id2 regulates microtubule (MT) dynamics through modulating acetylation of α-tubulin lysine 40 (αK40) in the neurons. Surprisingly, we found that in the postmortem brain of AD patients and AD model mice brain, the relatively low levels of Id2 expression along with a reduced αK40 acetylation, implying the diminution of Id2 is correlated with AD progression. Forced expression of Id2 but not Id2-S14A augmented αK40 acetylation and restored MT stability facilitating axonal growth in the hippocampus of 5X-FAD despite elevated Sirt2 activity, suggesting that upregulation of Id2 may be a novel strategy to improve MT dynamics in neurodegenerative diseases in which MT disorganization is implicated in pathogenesis.

Since Id2 influences both actin and MT activities, it could be an essential coordinator of cytoskeletal dynamics. The contribution of αK40 acetylation to MT stability is well established, but this is the first study implicating Id2 in this association. We show that Id2 interacts with α-tubulin, especially when phosphorylated by Akt at S14 and augments αK40 acetylation by blocking the access of the Sirt2 to the K40 site, in turn enhancing αK40 acetylation. Thus, augmenting Id2 expression or S14 phosphorylation can enhance αK40 acetylation and promotes MT polymerization, representing a new mechanism for coordination of actin and MT dynamics.

The expression of Id2 is relatively high in developing neurons but is reduced postnatally [3, 5]. Conversely, Sirt2 expression is very low in the developing brain but accumulates in the postnatal cortex [15]. Considering that Id2 strongly interacts with Sirt2 and specifically interferes with Sirt2 activity but not HDAC6 activity (Figs. 4B, C and 5E, G), it may be a primary regulator of α-tubulin acetylation in neurons. Presumably, Id2 protects α-tubulin acetylation for proper neural morphogenesis during embryonic development when Sirt2 expression is low while brain enrichment of Sirt2 after development is complete, and reduced Id2 expression contributes to processes of brain aging. In accordance with high levels of Sirt2 in the aged brain, the deficits shown in both normal aged brain and AD are associated with reduced acetylated α-tubulin and abnormally high Sirt2 [15, 19, 26–28]. Indeed, in the postmortem brain of AD patients and the hippocampus of 5X-FAD mice, we found relatively high levels of Sirt2 expression with a decreased αK40 acetylation. However, Id2 expression was certainly declined in the brain of both human AD patients and 5X-FAD mice. Overexpression of Id2 into either cultured hippocampal neurons or hippocampal slice of 5X-FAD apparently enhanced αK40 acetylation and displayed much longer axon growth (Fig. 6E–G), suggesting that the potential of Id2 counteracts pathogenic Sirt2 activity. Thus, the balance between Id2 and Sirt2 at different stages of brain development may be crucial for the regulation of αK40 acetylation and some neurodegenerative disorders may stem from an imbalance between Id2 and Sirt2, altering MT stability. While these signaling pathways require much further study, our result suggests that reactivation of Id2 signaling may represent an innovative strategy to modulate MT dynamics for neural function during the progression of AD.

## Materials and methods

### Culture of rat primary hippocampal neurons and cell lines

The hippocampi were removed from the brains of E18 rat embryos and placed in 15 ml tubes on ice containing 3 ml Hanks’ balanced salt solution (HBSS). Papain (20 mg per ml HBSS) was added and the samples incubated for 10 min at 37 °C. Digestion was stopped by washing the hippocampi twice with 5 ml HBSS containing 10% fetal bovine serum (FBS). Then, 3 ml of Neurobasal medium (NB, Invitrogen 21103-049)/B27 (Invitrogen 17504-044) was added, and the tissue was dissociated gently triturating through fire-polished Pasteur pipettes. The cell suspension was diluted to 10 ml with NB/B27 and then filtered through a 40 or 70 μm strainer. Cells were centrifuged at 2000 rpm for 2 min and resuspended in 5 ml NB/B27. Cells were maintained at 37 °C under a humidified 5% CO_2_ atmosphere. HEK293T cells (ATCCCRL-3216; PRID:CVCL_0063) and PC12 cells (ATCCCRL-1721; PRID:CVCL_0481) were obtained from ATCC. ATCC performs authentication and quality control tests on all distributions. All cell lines were authenticated periodically by cell morphology, growth curve analysis, and mycoplasma detection using a Mycoplasma detection kit (Roche) according to the ATCC cell line verification test recommendations. HEK293T cells were cultured in Dulbecco’s modified Eagle’s medium (DMEM) supplemented with 10% FBS and 100 U penicillin/streptomycin at 37 °C under a 5% CO_2_ atmosphere. PC12 cells were maintained in DMEM with 10% FBS, 5% horse serum, and 100 U of penicillin/streptomycin and maintained under the same conditions.

### Antibodies, small interfering (si)RNAs, and chemicals

Anti-Akt (cat. 2938s) and anti-p-Akt (S473, cat. 4060s) antibodies were acquired from Cell Signaling (Danvers, MA, USA), anti-α-tubulin (sc-8035), -β-actin (cat. sc-47778), -GFP (cat. sc-833s), -GST (cat. sc-138), -HA (cat. sc-7392), -Id2 (cat. sc-398104, -489), and -Sirt2 (cat. sc-28298) antibodies from Santa Cruz Biotechnology (Dallas, TX, USA), anti-FLAG (cat. F 1804), -Sirt2 (cat. S 8447), -acetylated tubulin (cat. T 7451), and -Tuj (cat. T 2200) antibodies from Sigma-Aldrich (St. Louis, MO, USA), anti-β-tubulin (cat. 801202) antibody from BioLegend (San Diego, CA, USA), and anti-NeuN (cat. ABN 78) antibody from Merck Millipore (Billerica, MA, USA). Alexa Fluor 555 Phalloidin, Alexa Fluor 546 goat anti-rabbit, and Alexa Fluor 488 goat anti-mouse secondary antibodies were obtained from Molecular Probes (Eugene, OR, USA). Anti-HSP70 was obtained from Abcam (Cambridge, MA, USA). The siRNA for silencing Id2 (5′-GAGCUUAUGUCGAAUGAUAUU-3′) and the siRNA for silencing Sirt2 (5′-AATCTCCACATCCGCAGGCAT-3′) were obtained from Genolution (Republic of Korea). Akt inhibitor VIII and nicotinamide adenine dinucleotide hydrate (NAD^+^) were obtained from Sigma (St. Louis, MO, USA).

### Construction of recombinant DNA and viral delivery system

A series of Id2 transcripts (WT, S14A, S14D) were cloned into the N-RFP vector. Fragments of the α-tubulin cDNA were cloned into the pcDNA-GST vector and fragments of Sirt2 cDNA were cloned into the pEGFP-C2 vector. For the expression and purification of Id2–GST fusion proteins, various Id2 constructs were cloned into the bacterial pGEX 4 T-1 vector. pLenti-si-Id2–GFP, pLenti-Id2–GFP, and pLenti-Sirt2–RFP were packaged by co-transfection with the psPAX2 lentiviral packaging plasmid and the vesicular stomatitis virus envelope glycoprotein-expressing pMD2.G plasmid in 293T cells using the Neon Transfection System (Thermo Fisher Scientific Inc.). The culture supernatant was harvested after 72 h, and the lentiviral particles were concentrated using a Beckman ultracentrifuge with SW41Ti rotor. The concentrated virus was resuspended in phosphate-buffered saline (PBS), aliquoted, and stored at −80 °C. To generate AAV2 constructs, Id2 WT, and S14A were inserted into the AAV2-IRES-GFP vector and packaged in the 293 AAV2 cell line for production of high-titer AAV2 (Cell Biolabs, Inc., CA, USA). The AAV2 packaging service was provided by KIST (Korea Institute of Science and Technology, Seoul, Republic of Korea).

### Co-immunoprecipitation and in vitro binding assays

For co-immunoprecipitation (co-IP), cells were rinsed with PBS and lysed in buffer containing 50 mM Tris-Cl, pH 7.4, 150 mM NaCl, 1 mM EDTA, 0.5% Triton X-100, 1.5 mM Na_3_VO_4_, 50 mM sodium fluoride, 10 mM sodium pyrophosphate, 10 mM β-glycerophosphate, 1 mM phenylmethylsulfonyl fluoride (PMSF), and protease cocktail (Calbiochem, San Diego, CA). Cell lysates (0.5 to 1 mg of protein) were mixed with primary antibody and protein A/G beads and incubated for 3 h at 4 °C with gentle agitation. The beads were then washed in lysis buffer and analyzed by immunoblotting. For GST pull-down assays, cells were rinsed with PBS and lysed in the same buffer described above. Cell lysates (0.5–1 mg of protein) were mixed with glutathione–sepharose beads and incubated for 3 h at 4 °C with gentle agitation. The beads were then washed in lysis buffer mixed with 2× SDS sample buffer, boiled, and analyzed by immunoblotting.

### Immunofluorescence

Cells grown on coverslips in 24-well plates were fixed in 4% paraformaldehyde (PFA) for 10 min, permeabilized in PBS containing 0.25% Triton X-100 for 10 min, and blocked in 1% bovine serum albumin (BSA) for 30 min. Cells were immunostained using primary antibodies and the appropriate Alexa Fluor 546-conjugated goat anti-rabbit or Alexa Fluor 488-conjugated goat anti-mouse secondary antibody. Immunostained images were acquired using a laser scanning confocal microscope (LSM 710, Carl Zeiss, Germany). The confocal microscope was controlled using ZEN software and the acquisition was performed in the Research Core Facility, LARC.

### In vitro deacetylase assay

HEK293T cells were transfected with Flag–Sirt2 or co-transfected with GFP–Id2 and Flag–Sirt2. For co-IP, cells were rinsed with PBS and lysed in the buffer used for co-IPs as described. Cell lysates (0.5–1 mg of protein) were mixed with anti-Flag antibody and protein A/G beads and incubated for 3 h at 4 °C with gentle agitation. The beads were then washed in deacetylase buffer (50 mM Tris-HCl, pH 9.0, 4 mM MgCl_2_, 0.2 mM DTT) and resuspended in deacetylase buffer containing 50 μg un-transfected HEK293T cell lysate and 1 mM NAD^+^. Precipitates were incubated for 2 h at room temperature with constant agitation. Reactions were stopped by adding SDS sample buffer. Beads were pelleted by centrifugation at 14,000 rpm for 10 min and analyzed by immunoblotting.

### In vitro tubulin polymerization assay

HEK293T cells were transfected with GST–Id2, harvested, and lysed in low salt buffer (20 mM Tris-HCl pH 6.8, 1 mM MgCl_2_, 2 mM EGTA, 0.5% NP-40, 1× protease inhibitor cocktail). Lysates were then centrifuged at 25,000 × *g* for 30 min at room temperature, and the supernatant was separated from the pellet. The pellet was resuspended in low salt buffer and sonicated. Equal volumes of supernatant and pellet samples were separated by SDS-PAGE and analyzed by immunoblotting. Tubulin polymerization assays were conducted using the CytoDYNAMIX Screen 03 assay system (Cytoskeleton, Inc.) following the manufacturer’s instructions. Tubulin (>99% pure) was reconstituted to 3 mg/mL in G-PEM buffer containing 80 mM PIPES, 2 mM MgCl_2_, 0.5 mM EGTA, 1 mM GTP (pH 6.9), and 15% glycerol in the absence or presence of the indicated compounds at 4 °C. The mixture was added to each well of a prewarmed 96-well plate and exposed to test compounds at varying concentrations (0.1–10 μM/L). The absorbance at 340 nm was recorded every 60 s for 1 h at 37 °C using a Bio-Rad xMark Microplate Absorbance Spectrophotometer (Bio-Rad, Hercules, CA). Dose–response curves were plotted using Prism 7 (Graphpad Software, Inc.).

### Proximity ligation assay (PLA)

PC12 cells were transfected with si-Id2, -Sirt2 using neon transfection system (Invitrogen) and seeded onto 12 mm glass coverslips in 24-well plates. The cells were fixed with 4% PFA. Primary antibodies used were anti-tubulin (rabbit, cat. 801202), anti-Id2 (mouse, cat. sc-389104), and mouse anti-tubulin (cat. T 2200), anti-Sirt2 (rabbit, cat. S 8447). Following the manufacturer’s protocol, secondary antibodies (anti-rabbit PLUS probe and anti-mouse MINUS probe, Sigma) were incubated with the cells, and following the Duolink protocol, if the two proteins were sufficiently close, rolling circle amplification was triggered by the subsequent additions. Amplified DNA was detected by a specific oligonucleotide that was labeled with a red fluorescent. Cells were analyzed via a confocal microscope (LSM 710, Carl Zeiss, Germany).

### Hippocampal slice culture

Hippocampal slice cultures were prepared from P20 mouse brains. Briefly, 280-mm-thick slices were obtained by vibratome sectioning (Leica VT1200, Leica Biosystems) in chilled MEMp [50% (vol/vol) minimum essential medium (MEM), 25 mM HEPES, and 2 mM glutamine without antibiotics, adjusted to pH 7.2–7.5 with 1 M NaOH]. The slices were transferred onto semi-porous membrane inserts (Millipore, 0.4 μm pore diameter, Schwalbach, Germany). Intact slices were cultured at 37 °C under a 5% CO_2_ atmosphere in MEMi [50% (vol/vol) MEM, 25 mM HEPES, 25% (vol/vol) HBSS 25% (vol/vol) heat-inactivated horse serum, 2 mM glutamine, 1 ml penicillin/streptomycin solution, and 0.044% (vol/vol) NaHCO_3_, adjusted to pH 7.2–7.3 with 1 M NaOH]. The medium was changed every other day. The hippocampal slices were infected with AAV after 7 days in vitro (DIV) and cultured for an additional 14 days before fixation in 4% PFA.

### Mice

This study was reviewed and approved by the Institutional Biosafety Committee (IBC) of Sungkyunkwan University School of Medicine (SUSM) (code 19-1-3-1) and the Institutional Animal Care and Use Committee (IACUC) of SUSM (code 19-3-15-1). SUSM is an Association for Assessment and Accreditation of Laboratory Animal Care International (AAALAC)-accredited facility (No. 001004) and abides by Institute of Laboratory Animal Resources (ILAR) guidelines. All experimental procedures were conducted following Sungkyunkwan University IACUC guidelines.

### Human tissue samples

Inferior parietal lobule specimens from the brains of patients with AD and individuals without dementia collected by the University of Kentucky Alzheimer’s Disease Center Autopsy Program were used for this study. The postmortem brain tissue samples were acquired under an IRB protocol approved by the University of Kentucky School of Medicine through an approved Institutional Review Board according to institutional guidelines. All patients with AD met the clinical and neuropathological diagnostic criteria for AD. Control subjects had no history or neuropathological symptoms of brain disorder. Informed consent was obtained from all subjects.

### Statistical analysis

Data are expressed as mean ± SEM of triplicate measurements from three independent experiments. Statistical analysis was performed using Sigmaplot Statistical Analysis Software (Systat Software, San Jose, CA, USA). All studies were performed in a blinded manner. Statistical significance was defined by Student’s *t*-test, one-way analysis of variance (ANOVA), or two-way ANOVA as indicated using GraphPad Prism 7 (GraphPad Software, Inc., San Diego, CA). A *p*-value < 0.05 (two-tailed) was considered statistically significant for all tests.

## Supplementary information

Supplemental information

Supplementary Figure 1

Supplementary Figure 2

Supplementary Figure 3

Supplementary Figure 4

## Data Availability

All data generated or analyzed during this study are included in this published article.
