# Correction to: Inhibitor of DNA binding 2 (Id2) mediates microtubule polymerization in the brain by regulating αK40 acetylation of α-tubulin

**DOI:** 10.1038/s41420-021-00673-z

**Published:** 2021-10-08

**Authors:** Taegwan Yun, Hyo Rim Ko, Dong-Gyu Jo, Kye Won Park, Sung-Woo Cho, Jihoe Kim, Jee-Yin Ahn

**Affiliations:** 1 Department of Molecular Cell Biology, University School of Medicine, 16419 Suwon, Korea; 2 Single Cell Network Research Center Sungkyunkwan, University School of Medicine, 16419 Suwon, Korea; 3 Department of Health Science and Technology, Samsung Advanced Institute for Health Science and Technology, Sungkyunkwan University, 06351 Seoul, Korea; 4 School of Pharmacy, Sungkyunkwan University, 16419 Suwon, Korea; 5 Department of Food Science and Biotechnology, Sungkyunkwan University, 16419 Suwon, Korea; 6 Department of Biochemistry and Molecular Biology, University of Ulsan, College of Medicine, 05505 Seoul, Korea; 7 Department of Medical Biotechnology, Yeungnam University, 38541 Gyeongsan, Republic of Korea; 8 Samsung Biomedical Research Institute, Samsung Medical Center, 06351 Seoul, Korea

**Keywords:** Microtubules, Phosphorylation

Correction to: *Cell Death Discovery* 10.1038/s41420-021-00652-4, published online 21 September 2021

The original version of this article unfortunately contained an error in Dong-Gyu Jo’s name. The authors apologize for the error. The original article has been corrected.

