# Supplementary material for: Inhibitor of DNA binding 2 (Id2) mediates microtubule polymerization in the brain by regulating αK40 acetylation of α-tubulin
Source: Cell Death Discov. 2021 Sep 21;7:257. doi: 10.1038/s41420-021-00652-4 (PMC8455547; doi:10.1038/s41420-021-00652-4)
Supplement: Supplementary file 1 — Supplemental information [file 41420_2021_652_MOESM1_ESM.doc]

**Supplementary information**

**Supplementary Figure legends**

Supplementary Figure 1.

Coomassie brilliant blue staining of purified Id2 WT, S14A, S14D, and GST control protein.

Supplementary Figure 2.

(A and B) PC12 cells were transfected with scr, si-Id2, or si-Sirt2. Confocal images were shown PLA staining without primary antibodies (A) and negative control using mouse IgG and rabbit IgG antibodies as primary antibodies (B). Nuclei were stained by DAPI staining (blue). Scale bar 5 μm.

Supplementary Figure 3.

(A) The paraffin section was stained with anti-Aβ antibody (green). Scale bar 200 µm. (B) The paraffin-embedded section was immunostained with anti-sirt2 (red) and anti-acetyl- α-tubulin (green). Profile image shows the intensity of acetyl-α-tubulin (green), Sirt2 (red). (C) Control and 5XFAD hippocampal neurons were cultured for 18 days. The fixed neuron was immunostained with anti-acetyl-α-tubulin (green) and Tubulin (red). Scale bar 20 μm. Quantification of fluorescence intensity of Acetyl-α-tubulin and α-tubulin are shown right. (D) The Hippocampal neuron was cultured for 6 days. The neurons were transfected with control, Id2 (WT), and S14A on DIV 5. After 48 hrs transfection, the neurons were fixed with 4% paraformaldehyde and subjected to immunostaining with anti-Tau antibody (blue) and anti- acetyl-α-tubulin antibody (green). Quantification of fluorescence intensity and axon length is shown on the right. Scale bar 20 μm. *p<0.05 **p<0.005. (E) The Hippocampal neuron was cultured for 7 days. The neurons were fixed with 4% paraformaldehyde and subjected to immunostaining with anti-Tau antibody (green) and anti- Id2 antibody (red). Scale bar 10 μm. Profile image shows the intensity of Tau (green) and Id2 (red). Neurite Tip is magnified in the expanded box to the right. Scale bar 2 μm.

Supplementary Figure 4.

(A and B) (A) The control and 5X-FAD brain slices were cultured for 28 days. The slices were fixed with 4% paraformaldehyde on DIV 28 and subjected to immunostaining with anti-Aβ antibody (green). Scale bar 20 μm. (B) Profile image shows the intensity of NeuN (blue), AAV (green), and Acetyl-α-tubulin (red).
