# Supplementary figures and images for: Inhibitor of DNA binding 2 (Id2) mediates microtubule polymerization in the brain by regulating αK40 acetylation of α-tubulin
Source: Cell Death Discov. 2021 Sep 21;7:257. doi: 10.1038/s41420-021-00652-4 (PMC8455547; doi:10.1038/s41420-021-00652-4)

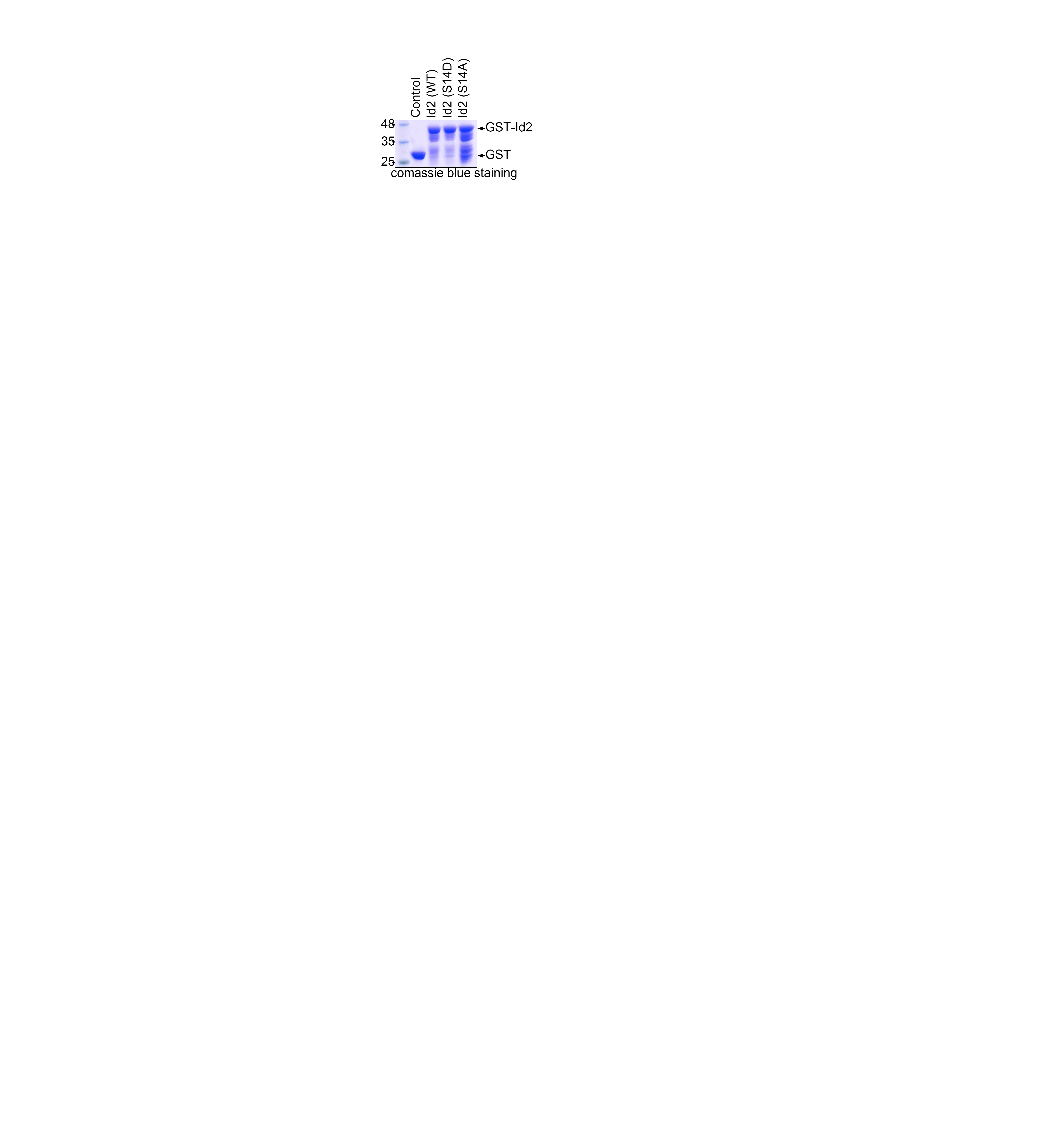

Supplement: Supplementary file 2 — Supplementary Figure 1 [file 41420_2021_652_MOESM2_ESM.tif]

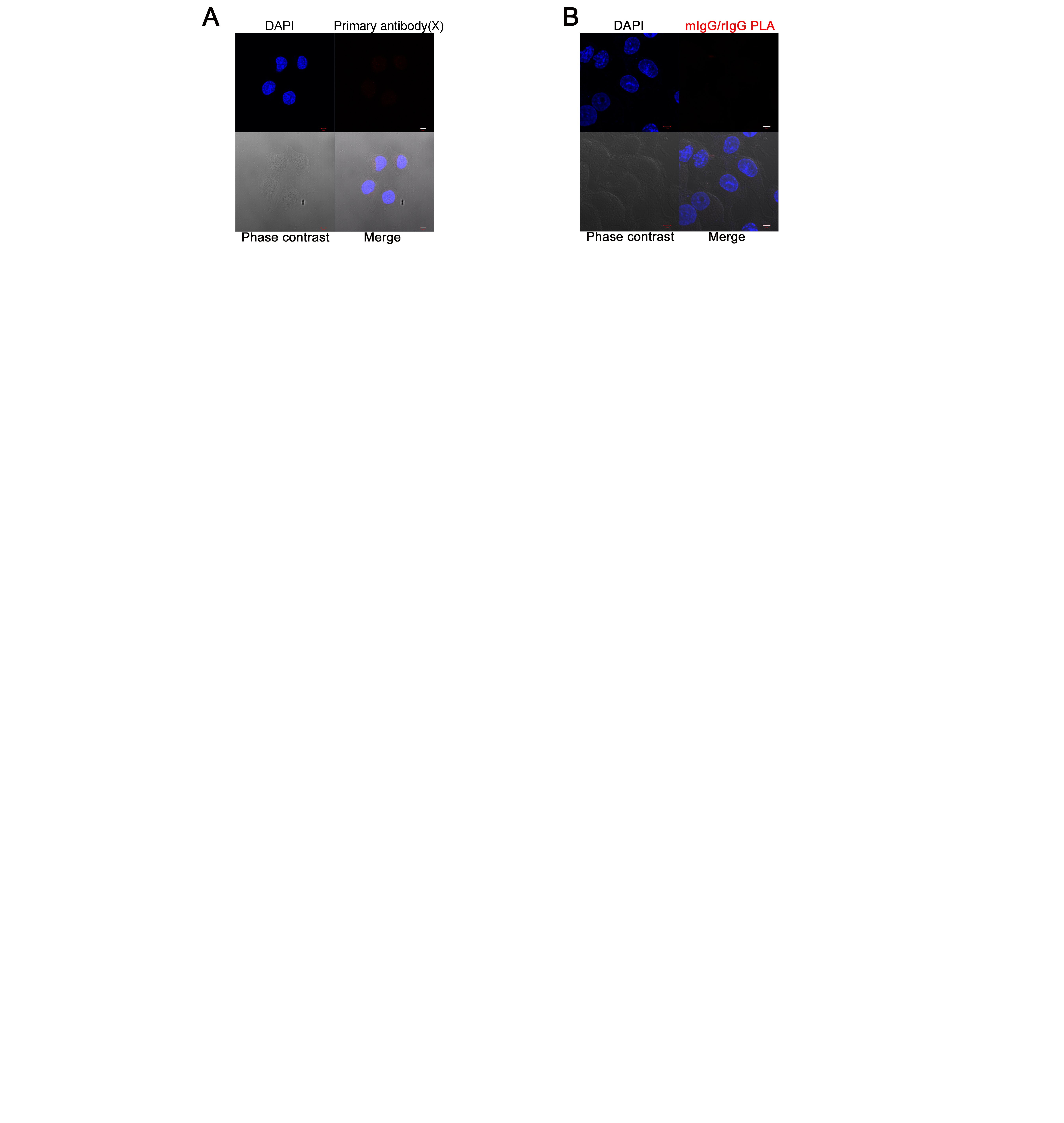

Supplement: Supplementary file 3 — Supplementary Figure 2 [file 41420_2021_652_MOESM3_ESM.tif]

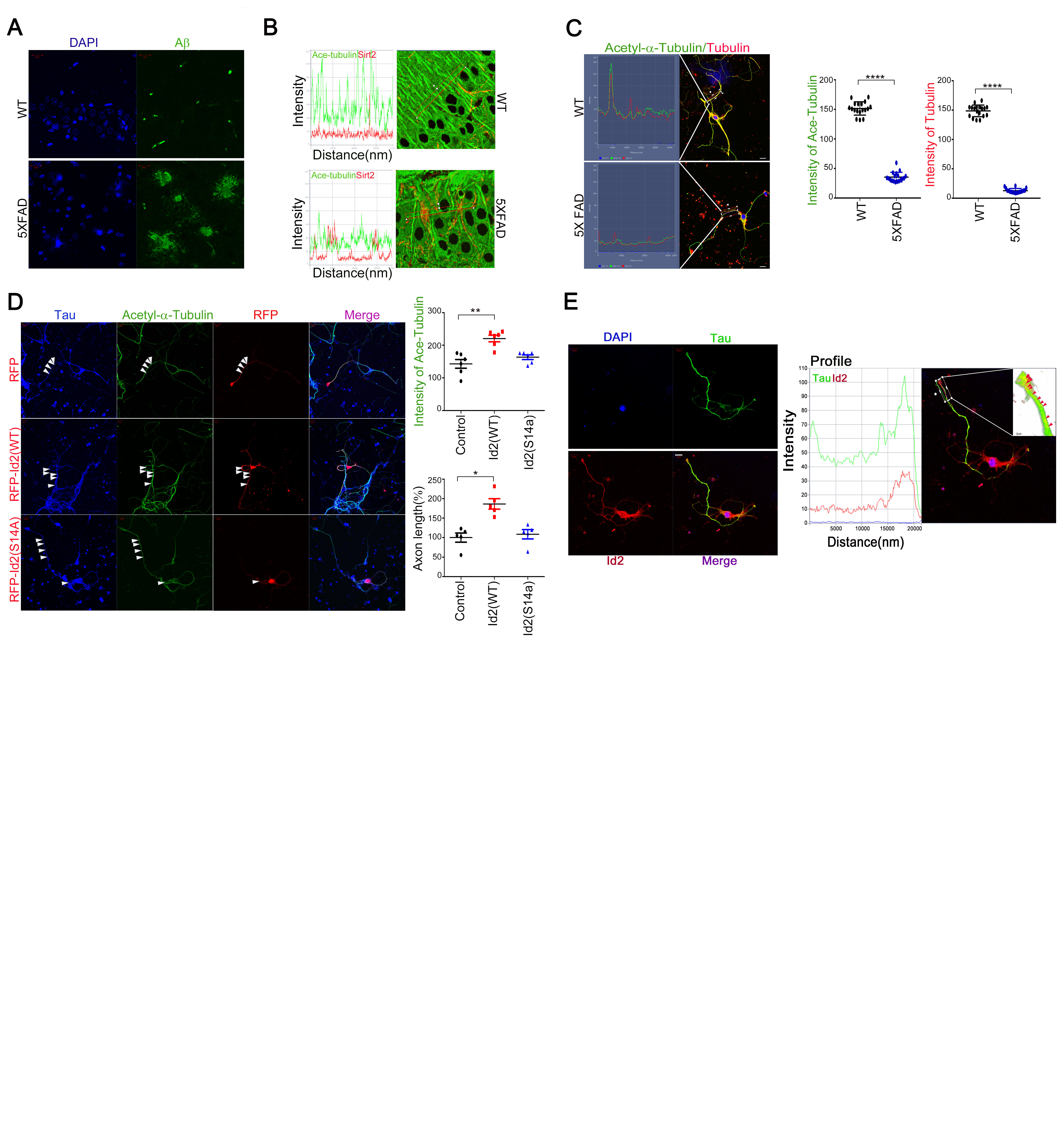

Supplement: Supplementary file 4 — Supplementary Figure 3 [file 41420_2021_652_MOESM4_ESM.tif]

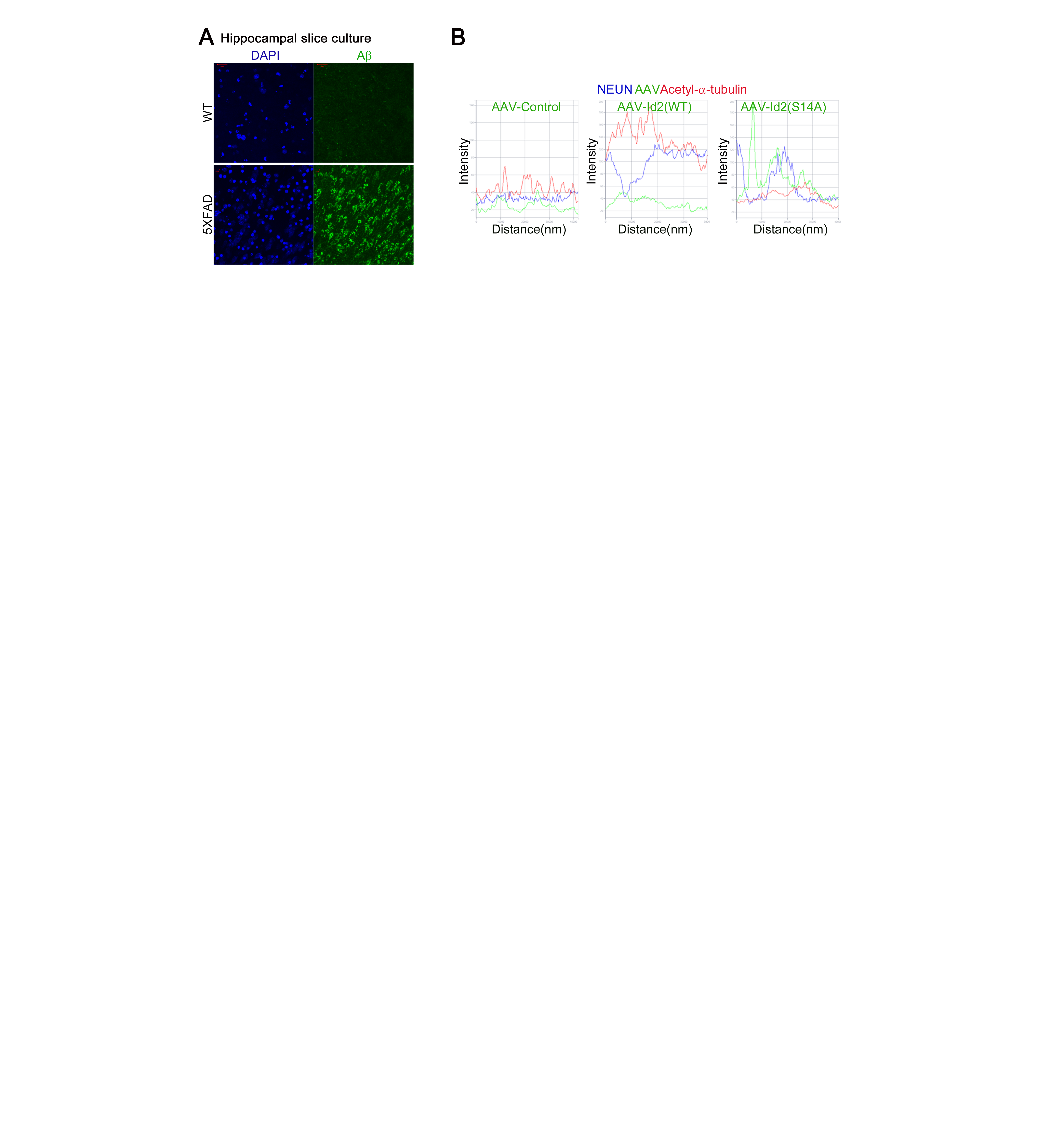

Supplement: Supplementary file 5 — Supplementary Figure 4 [file 41420_2021_652_MOESM5_ESM.tif]
